# Comparison of the Effectiveness of Warmed Versus Room Temperature Intravenous Fluids Administration to Prevent Intraoperative Heat Loss in Anaesthetised Calves Undergoing Umbilical Herniorrhaphy

**DOI:** 10.1002/vms3.70096

**Published:** 2024-10-26

**Authors:** Sitkican Okur, Latif Emrah Yanmaz, Mumin Gökhan Senocak, Ayse Golgeli, Ferda Turgut, Omer Tarik Orhun, Yakup Kocaman, Ugur Ersoz

**Affiliations:** ^1^ Department of Surgery Faculty of Veterinary Medicine Atatürk University Erzurum Turkey; ^2^ Department of Surgery Faculty of Veterinary Medicine Mehmet Akif Ersoy University Burdur Turkey

**Keywords:** anaesthesia, calves, hypothermia, rectal temperature, warm

## Abstract

**Background:**

Warmed intravenous (IV) fluids administration to prevent hypothermia provide controversial results in humans, cats and dogs, but its effect on calves is unknown.

**Objectivxe:**

To evaluate the effectiveness of warmed IV fluids administered to prevent intraoperative heat loss in anaesthetised calves undergoing umbilical herniorrhaphy.

**Methods:**

Thirty Simmental breed calves (aged 10–30 days) were randomly divided between two equal groups, wherein the infusion fluid (Ringer's lactate, 5 mL/kg/h) was administered either at room temperature (Group RoT) or warmed (Group WF). Pulse rate (PR), respiratory rate (*f*_R_), peripheral haemoglobin oxygen saturation (SpO_2_) and rectal temperature (RT) were recorded immediately after the onset of anaesthesia induction (T_0_) at T_5_, T_10_, T_15_, T_30_, T_45_ and T_60_. Duration of anaesthesia, surgery time and recovery scores were also noted.

**Results:**

The PR, RT and *f*_R_ values showed no significant difference between groups over time (*p* > 0.05). There was no significant difference in duration of anaesthesia, surgery time or recovery score between groups (*p* > 0.05).

**Conclusions:**

The findings of the current study suggest that warmed IV fluid as the warming method did not prevent intraoperative hypothermia in calves. A constant‐rate infusion of warmed fluid (5 mL/kg/h) is insufficient to prevent intraoperative hypothermia in calves.

## Graphical abstract

Hypothermia is one of the major complications of surgery. Warmed fluids are often used in humans to prevent hypothermia. However, their efficacy in preventing hypothermia in dogs and cats is a topic of controversy. Using warmed fluids in calves does not effectively prevent hypothermia. Thus, further warming techniques should be used during umbilical herniorrhaphy in calves, in addition to administering warmed fluids

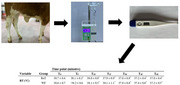

## 1 Introduction

Hypothermia is defined as a body temperature (BT) 1–3°C below normal (Chiang, Hopper, and Mellema 2011) and has been observed in more than 97% and 84% of anaesthetised cats and dogs, respectively (Redondo, Suesta, Gil, et al. 2012; Redondo, Suesta, Serra, et al. 2012). The main causes of significantly reduced BT during anaesthesia are decreased heat production and increased heat loss, with the latter occurring via one or more of the following mechanisms: preoperative skin disinfection (evaporation), contact with cold table surfaces (conduction) and open body cavities (radiation and evaporation) (Haskins 2015; Tan et al. 2004). Since the BT regulation of newborn calves is metabolically immature, the calves must be protected from heat loss while under anaesthesia (Hill et al. 2016; Lin and Walz 2014).

Hypothermia may increase the risk of numerous adverse effects, such as the delayed healing of surgical wounds, delayed recovery from anaesthesia, coagulopathy, arrhythmia, hypotension, respiratory depression, prolonged hospitalisation and death (Tan et al. 2004; Lee et al. 2014; Jourdan et al. 2017). Therefore, protection against anaesthesia‐induced hypothermia is crucial in veterinary and human medicine. Several passive and active warming methods are already widely used to prevent hypothermia in cats (Sakata et al. 2020) and dogs (Tan et al. 2004). Prewarmed intravenous (IV) fluids are a common method in veterinary medicine (Dix et al. 2006). Previous studies have reported that the constant rate infusion (CRI) of heated fluids successfully prevents intraoperative hypothermia in human patients (Xu et al. 2010; Campbell et al. 2015). A study carried out on cats reported that warmed IV infusions significantly influence the decrease of heat loss while under anaesthesia (Steinbacher et al. 2010). However, another study found no beneficial thermodynamic effects when comparing the use of prewarmed and room temperature IV fluids in cats (Jourdan et al. 2017). General anaesthesia is suggested for performing the surgical procedures in young calves (Ducharme, Desrochers, and Freeman 2017). BT of cattle rarely decreases by more than 0.5°C under anaesthesia unless a major body cavity is opened. A large surface area to mass ratio, a low percentage of white adipose tissue and reduced shivering thermogenesis capabilities predispose calves to heat loss (Lin and Walz 2014). Maintaining the extremities warm (e.g., by wrapping the limbs in warm towels or bubble wrap) and employing a forced warm air system (e.g., 3 M Bair Hugger Therapy) over the bovine surgical patient during surgery can be an effective way to prevent hypothermia (Perkins et al. 2017). However, there are no reports of the effectiveness of these methods in calves.

To our knowledge, no studies have reported the effect of prewarmed fluids administered by CRI as a method of active warming during general anaesthesia in calves. For this reason, the purpose of this study was to evaluate the effect of warmed IV for the prevention of hypothermia during general anaesthesia in calves undergoing umbilical herniorrhaphy. Our hypothesis was that the BT of calves receiving warmed IV fluids would be higher than that of calves that received room temperature IV fluids while under general anaesthesia.

## 2 Materials and Methods

This prospective study was approved by the Atatürk University Local Board of Ethics Committee (168/2020). Thirty Simmental breed calves aged 10–30 days were presented to the Atatürk University Animal Hospital between February and December 2022 for umbilical herniorrhaphy. All calves were considered healthy based on complete blood count and physical examination, according to American Society of Anaesthesiologists (ASA) grade II. Written informed consent was obtained from the calves' owners before or at the time of surgery. Food, but not water, was withdrawn from the calves for 3 h before the beginning of the surgery. Exclusion criteria were preoperative rectal temperature (RT) >39.5°C or <38.5°C and calves aged >30 or <10 days.

Thirty Simmental calves were divided into two equal groups using block randomisation (block size 2, random.org). The first group (*n* = 15) received intraoperative IV fluids at room temperature (∼23°C; Group RoT) and the second group (*n* = 15) received intraoperative IV in‐line fluid warmed with a fluid warmer (Shenzhen Hawk, 838SI, Shenzhen, China) set at 43°C (Group WF). The fluid warmer was attached to a 60 cm extension tube in both groups. During the study, all calves' fluid replacement was administered by CRI of the balanced electrolyte fluids (Ringer's lactate, 5 mL/kg/h) (Walz 2014) using an infusion pump (Birset, Type CF IPX4, Ankara, Turkey). To evaluate the temperature of the fluid warmer, the temperature of the fluid was measured by using a thermal camera (IR Flexcam S; Infrared Solutions Incorporated, Plymouth, MN, USA) at the stage when the fluid leaves the fluid warmer and just before it enters the IV catheter in the patient. The fluid warmer, infusion pump and thermal camera were calibrated before use.

All surgery procedures were performed in the same operating room and by the same surgeon who was unaware of group allocation. Before starting the surgery, the room temperature was measured in five different locations with a room temperature measuring device (Diwu, HTC‐1, Wenzhou, China), and an average room temperature was calculated. All animals were acclimated to the operating room's environmental temperature for 30 min before the surgery. The calves underwent the same anaesthesia protocol, consisting of intramuscular 40 µg/kg detomidine (Domosedan, Zoetis, NJ, USA) and 5 mg/kg intramuscular ketamine (Ketasol, Richter Pharma, Wels, Austria). Lidocaine hydrochloride (2%, Aritmal, Osel İlaç, Beykoz, Istanbul) was used to provide circular infiltration analgesia around the surgical site. A maintenance dose of ketamine (2 mg/kg) was intravenously administered when spontaneous movements occurred during surgery. For CRI, a venous catheter (22G, Bıcakcılar IV catheter, Istanbul, Turkey) was inserted into the marginal vein of the ear. All calves received 22 mg/kg cefazoline (Cefamezin, Eczacibasi, Istanbul, Turkey) just before the beginning of surgery and were placed in dorsal recumbency on a heated stainless steel surgical table (set at 37.5°C) covered with cotton surgical drapes. No additional heating procedure was performed throughout the study. Following surgery, 1.5 mg/kg IV flunixin meglumine (Flumeglin, Teknovet, Istanbul, Turkey) was administered to all calves. During anaesthesia, pulse rate (PR) and peripheral haemoglobin oxygen saturation (SpO_2_) were continuously monitored with a clip‐on type transmittance probe placed at the base of the tongue, and RT was measured with the insertion of a fibre optic temperature sensor (15 cm) into the rectum (Comen C80‐V, Shenzhen, China). The temperature sensor's accuracy was tested within 0.1°C using a water bath before each surgery day. Respiratory rate (*f*_R_) was evaluated as observation of chest movement. All measurements were recorded immediately after the onset of anaesthesia induction (T_0_), at T_5_ (+5 min), T_10_ (+10 min), T_15_ (+15 min), T_30_ (+30 min), T_45_ (+45 min) and T_60_ (+60 min).

The duration of anaesthesia, surgery time and recovery scores were also recorded. Duration of anaesthesia was defined as the time from the onset of spontaneous recumbency after intramuscular detomidine and ketamine to the calves' first spontaneous movement after the completion of surgery. The duration of surgery was defined as the beginning of the incision to the last skin suture. The recovery score was evaluated at the end of the surgery using a modified scoring system previously described by Alonso et al. (2020). Scores assigned to each parameter were as follows: 1 = one attempt, little to no ataxia; 2 = one or two attempts, some ataxia; 3 = more than two attempts, quiet recovery; 4 = more than two attempts, moderate recovery; 5 = more than two attempts, excitation; 6 = very bad recovery, high risk of injury. All recovery scores were assessed by the same researcher, who was unaware of group allocation.

Power analysis (PS‐Power and Sample Size Calculation, Version 3.1.2, Vanderbilt University, TN, USA) was performed to estimate the minimum sample size needed in each group. The calculation was based on data previously collected in Animal Hospital. The sample size was calculated to determine a difference of 2°C (standard deviation of ±0.5°C) in the BT between groups, with a level of significance of 0.05 and a power of 90%.

The normality of the data was tested before analysis using the Shapiro–Wilk test. Data expressed as mean ± standard deviation and the repeated measures analysis of variance (ANOVA), following the Bonferroni post hoc test were employed for RT, PR, *f*_R_ and SpO_2_ measurement. To determine differences in these parameters at each time point, a two‐way ANOVA was used, and the Bonferroni test was used to assess differences between groups at these times. Dunnett's test was performed to compare the other time points to T_0_ within each group. Non‐repeated data were tested using a Student's *t*‐test. Nonparametric data were analysed using a Mann–Whitney *U* test. All data analyses were performed with the SPSS Version 25.0 (IBM Company, SPSS Inc., IL, USA) and values of *p* < 0.05 were considered statistically significant.

## 3 Results

All calves recovered from anaesthesia without complication. No adverse effects (local inflammation or venous pain) were encountered in the WF and RoT groups during the postoperative 3 h.

There was no significant difference in mean age (17 ± 4 vs. 19 ± 6 days), sex (female [*n*] = 6, male [*n*] = 9 vs. female [*n*] = 5, male [*n*] = 10) or bodyweight (38.7 ± 5.6 vs. 40.1 ± 7.2 kg) between the groups RoT and WF, respectively (*p* < 0.05). The baseline RT measurements the morning of surgery were not significantly different between groups (Group RoT: 39.1 ± 0.6°C vs. Group WF: 39.0 ± 0.2°C, *p* = 0.87). During all procedures, the room temperature between the RoT (23.2 ± 0.9°C) and WF groups (24.1 ± 0.5°C) was not statistically significant (*p* = 0.62).

Table 1 depicts the mean RT, PR, *f*_R_ and SpO_2_ values for Groups RoT and WF at various times. No significant differences emerged between the groups at T_0_ means of RT, PR, *f*_R_ and SpO_2_ values (*p* = 0.42, *p* = 0.09, *p* = 0.54 and *p* = 0.11, respectively, Table 1). Moreover, no significant differences in RT, PR and *f*_R_, values were observed between groups over time (*p* > 0.05). SpO_2_ values were significantly different between groups at T_15_ (*p* = 0.03). In the RoT group, RT progressively decreased from T_5_ to T_60_, and in the WF group, RT progressively decreased from T_10_ to T_60_ (*p* < 0.05; Table 1). There was no significant difference in RT between the RoT and WF groups at the same time point (*p* > 0.05). In the WF group, at T_45_ and T_60_, one and six animals, respectively, showed RT measurements lower than 37°C, whereas in the RoT group, at T_45_ and T_60_, two and four animals, respectively, showed RT measurements lower than 37°C (*p* > 0.05).

**TABLE 1:** Effect of intravenous (IV) room temperature fluid (RoT, *n* = 15, 5 mL/kg/h) and warmed IV fluid (WF, *n* = 15, 5 mL/kg/h) groups on rectal temperature (RT), pulse rate (PR), respiratory frequency (*f*_R_) and arterial haemoglobin oxygen saturation (SpO_2_) in 30 calves undergoing umbilical herniorrhaphy. All measurements were noted immediately after induction of anaesthesia (T_0_) until 60 min (T_60_).

		Time point (min)						
Variable	Group	T_0_	T_5_	T_10_	T_15_	T_30_	T_45_	T_60_
RT (°C)	RoT	38.7 ± 0.4	38.1 ± 0.2^a^	38.0 ± 0.8^a^	37.9 ± 0.9^a^	37.6 ± 0.8^a^	37.2 ± 0.4^a^	37.0 ± 0.4^a^
	WF	38.6 ± 0.7	38.2 ± 0.6	38.1 ± 0.5^a^	38.1 ± 1.1^a^	37.8 ± 0.6^a^	37.4 ± 0.8^a^	37.2 ± 0.5^a^
PR (min^−1^)	RoT	103 ± 15	95 ± 9	90 ± 12	97 ± 13	90 ± 12	89 ± 9^a^	91 ± 10
	WF	99 ± 21	94 ± 12	93 ± 14	101 ± 18	87 ± 18	92 ± 15	88 ± 16
*f*_R_ (min^−1^)	RoT	40 ± 9	38 ± 4	33 ± 5^a^	37 ± 6	38 ± 9	31 ± 10	35 ± 9
	WF	38 ± 11	38 ± 10	36 ± 12	39 ± 4	34 ± 8	36 ± 9	35 ± 7
SpO_2_ (%)	RoT	91 ± 4	94 ± 4	95 ± 2	82 ± 6^a^, ^b^	94 ± 3	92 ± 4	95 ± 2
	WF	96 ± 2	94 ± 4	94 ± 3	90 ± 4^b^	88 ± 5^a^	91 ± 5	90 ± 1

*Note*: Data expressed as mean ± standard deviation.

^a^ Significantly different from baseline within the group (*p* < 0.05).

^b^ Significant difference between groups (*p* < 0.05).

There was no significant difference in duration of anaesthesia (73.2 ± 11.6 vs. 76.5 ± 13.2 min; *p* = 0.11), surgery time (51.2 ± 14.2 vs. 49.1 ± 10.5 min; *p* = 0.23) or recovery score (2.23 ± 1.40 vs. 2.04 ± 1.32) in both groups (*p* > 0.05; Table 2). Six calves in both groups were administered a maintenance IV 2 mg/kg ketamine (*p* > 0.05).

**TABLE 2:** Mean duration of anaesthesia, surgery time and recovery score values in intravenous (IV) room temperature fluid (RoT, *n* = 15) and warmed IV fluid (WF, *n* = 15) groups.

Group	Duration of anaesthesia (min)	Surgery time (min)	Recovery score
RoT	73.2 ± 11.6	51.2 ± 14.2	2.23 ± 1.40
WF	76.5 ± 13.2	49.1 ± 10.5	2.04 ± 1.32

*Note*: Data expressed as mean ± standard deviation.

The IV fluid line output temperature from the fluid warmer device was 39.8 ± 0.3°C and significantly decreased at 33.1 ± 0.6°C (*p* < 0.001) the IV fluid line entering the veins of the animals.

## 4 Discussion

This study investigated the effectiveness of warmed IV fluids in preventing hypothermia in calves. We found that warmed IV fluids (5 mL/kg/h) failed to prevent a decrease in BT during anaesthesia in calves undergoing umbilical herniorrhaphy.

The literature has described many active warming methods to prevent hypothermia in various species, such as bubble wrap, insulating blankets, electric and reflective blankets, warmed IV fluids, warm abdominal lavage, circulating heated water pads and warm air blankets (Tan et al. 2004; Campbell et al. 2015; Soto et al. 2014; Kibanda and Gurney 2012). However, surface heating methods typically fail to increase core temperature unless the temperature of the heating system is higher than the patients' core temperature (Pope 2003). Furthermore, the chance of adverse effects increases when the surface heating methods are performed at higher temperatures, with surface burns occurring in animals under prolonged exposure to heating pads set at 42°C (Pope 2003). In addition, the disruptive effects of anaesthesia on peripheral circulation in animals may enhance the risk of thermal damage from surface heating methods (Nawrocki, McLaughlin, and Hendrix 2005). Electric fluid warmers, meanwhile, follow a simple method of active core warming that involves passing an infusion set through a warming device. Such warmers are comparatively economical, easy to store because of their small size and quick and easy to set up.

Previous studies conducted on humans reported that preoperative and intraoperative administration of warmed fluids effectively prevents hypothermia (Campbell et al. 2015). However, in our study, warmed fluids did not effectively prevent hypothermia in calves undergoing umbilical herniorrhaphy. This discrepancy could be explained by small and/or neonatal animals' BT differing from humans. Small or neonatal animals may have elevated metabolic rates, leading to faster body heat loss. Some animal species have a tendency to decrease their BT, especially while under anaesthesia. Hence, warmed fluids in animals may not be as effective in avoiding hypothermia as in humans (Ducharme, Desrochers, and Freeman 2017).

In this study, the BT of the calves in both groups (RoT and WF) decreased about 2°C during the first hour of anaesthesia. This decrease in BT has been previously reported in cats (Jourdan et al. 2017), dogs (Redondo, Suesta, Gil, et al. 2012) and children (Xu et al. 2010) during anaesthesia. Physiological responses to hypothermia are often reduced in animals under general anaesthesia compared to when they are conscious (Murison 2001). Generally, anaesthetic drugs result in impaired hypothalamic thermostatic mechanisms, which reduce the hypothalamus's control over BT (Murison 2001). In addition, hypothermia is expected as a result of α_2_‐adrenoceptor agonist administration in animals owing to reduced heat production (decreased muscle activity) and the impact on thermoregulation (MacDonald, Scheinin, and Scheinin 1998).

The current study reported that warmed IV fluid, used as the active warming method to prevent or decrease hypothermia, failed in calves undergoing abdominal surgery compared with room‐temperature IV fluids administered under the same condition. This is in agreement with a previous report on cats (Jourdan et al. 2017). This failure to prevent hypothermia could be explained by one or more of the following mechanisms: heat loss along the IV line during its passage through the fluid infusion line (Soto et al. 2014), heat dissipation due to the fluid's slow flow rate (Lee et al. 2014) and there are limits to both the volume of fluids given and their rate of administration because of patient size and health status (Barthel and Pierce 2012). Previous in vitro studies (Chiang, Hopper, and Mellema 2011; Soto et al. 2014; Horowitz et al. 2004) suggested that the infused fluid should be administered at flow rates faster than at 60 mL/h to prevent hypothermia. Similarly, another study in cats reported that low rate of fluid administration (5 mL/kg/h) is not sufficient to prevent hypothermia (Jourdan et al. 2017). Furthermore, the findings in the present study indicated that infusing rate 5 mL/kg/h in calves was not also adequate to prevent decline in BT. A previous study has stated that the loss of heat between fluid line exiting of fluid warmer device and entering the vein is 12°C (Jourdan et al. 2017), which is consistent with our findings. Although the highest temperature setting (43°C) was used on the fluid warmer device, this was not enough to preclude hypothermia. This confirms previous study that states IV fluid warmer device has no effect on maintaining core BT in large breed dogs (Brady and Poppell 2020).

In our study, SpO_2_ levels of calves in both groups decreased at T_15_ and T_30_. The cause of this decrease is unclear. One potential reason for the fall in SpO_2_ levels might be attributed to calves' movements during surgery or inaccurate sensor readings. Another investigation in humans found that SpO_2_ levels might drop during movement because of changes in the optical path length through the tissue (Clarke, Chan, and Andy 2014).

There are some limitations to this study. Notably, the 60 cm IV fluid line between the warmer device and the calves potentially caused heat loss via convection and radiation as the fluid passed through the line. However, if the location of the warmer device is too close to the calves, it may result in thermal vascular injury. In addition, although core BT is considered the ‘gold standard’ of temperature assessment (Okur et al. 2022), with RT slightly lower in comparison (Southward et al. 2006), we did not measure it in this study.

In conclusion, the current study indicates that the administration of warmed IV fluid using a heating device fails to prevent or reduce the incidence of intraoperative hypothermia in calves undergoing umbilical herniorrhaphy while under general anaesthesia. For this reason, calves undergoing umbilical herniorrhaphy must be supported with additional warming methods.

## Author Contributions

**Sitkican Okur**: conceptualization, investigation, data curation, writing–original draft preparation, formal analysis. **Latif Emrah Yanmaz, Mumin Gökhan Senocak and Ayse Golgeli**: supervision, manuscript revision, methodology, investigation, visualisation. **Ferda Turgut, Omer Tarik Orhun, Yakup Kocaman and Ugur Ersoz**: manuscript revision, supervision, visualization. All author reviewed the results and approved the final version of paper.

## Ethics Statement

This prospective study was approved by the Atatürk University Local Board of Ethics Committee (168/2020).

## Conflicts of Interest

The authors declare no conflicts of interest.

### Peer Review

The peer review history for this article is available at https://www.webofscience.com/api/gateway/wos/peer-review/10.1002/vms3.70096.

## Data Availability

Data set and analyses are available from the corresponding author upon reasonable request.
